# Spontaneous Cytokine Production in Children According to Biological Characteristics and Environmental Exposures

**DOI:** 10.1289/ehp.0800366

**Published:** 2009-01-09

**Authors:** Camila Alexandrina Figueiredo, Neuza Maria Alcântara-Neves, Rafael Veiga, Leila D. Amorim, Vitor Dattoli, Lívia Ribeiro Mendonça, Samuel Junqueira, Bernd Genser, Mariese Santos, Lain Carlos Pontes de Carvalho, Philip J. Cooper, Laura Rodrigues, Maurício L. Barreto

**Affiliations:** 1 Instituto de Ciências da Saúde; 2 Instituto de Matemática and; 3 Instituto de Saúde Coletiva, Universidade Federal de Bahia, Salvador, Brazil;; 4 Centro de Pesquisas Gonçalo Moniz–FIOCRUZ, Salvador, Brazil;; 5 Universidad San Francisco de Quito, Quito, Ecuador;; 6 London School of Hygiene and Tropical Medicine, London, United Kingdom

**Keywords:** age, breast-feeding, cytokine profile, IL-10, SCAALA, sewage, sex, tap water

## Abstract

**Background:**

Environmental factors are likely to have profound effects on the development of host immune responses, with serious implications for infectious diseases and inflammatory disorders such as asthma.

**Objective:**

This study was designed to investigate the effects of environmental exposures on the cytokine profile of children.

**Methods:**

The study involved measurement of T helper (Th) 1 (interferon-gamma), 2 [interleukin (IL)-5 and IL-13], and the regulatory cytokine IL-10 in unstimulated peripheral blood leukocytes from 1,376 children 4–11 years of age living in a poor urban area of the tropics. We also assessed the impact of environmental exposures in addition to biological characteristics recorded at the time of blood collection and earlier in childhood (0–3 years before blood collection).

**Results:**

The proportion of children producing IL-10 was greater among those without access to drinking water [*p* < 0.05, chi-square test, odds ratio (OR) = 1.67]. The proportion of children producing IL-5 and IL-10 (OR = 10.76) was significantly greater in households that had never had a sewage system (*p* < 0.05, trend test).

**Conclusions:**

These data provide evidence for the profound effects of environmental exposures in early life as well as immune homeostasis in later childhood. Decreased hygiene (lack of access to clean drinking water and sanitation) in the first 3 years of life is associated with higher spontaneous IL-10 production up to 8 years later in life.

Host genetics, environmental exposures, and gene–environment interactions are likely to be important determinants in the development of human immunity (Ogra and Weliver 2008). Important environmental exposures that may affect immune development include early-life microbial colonization of the intestine and exposures to pathogens, parasites, and microbial products (Guarner 2006; Maizels and Yazdanbakhsh 2003; Strachan 1989). Such exposures are likely to be important for the normal development of a regulated immune response that maintains immune homeostasis under normal physiologic conditions (Ogra and Weliver 2008).

Latin America is undergoing rapid population change, including urbanization, migration, economic development, and adoption of a “westernized” lifestyle. Efforts to improve water supply, sanitation, waste collection, and other hygienic measures are common in the different Latin American countries, and it is not clear whether cleaning of a highly contaminated environment, from a microbiological viewpoint, may affect the development and maintenance of human immune homeostasis. A decline in infectious and microbial exposures in childhood may help explain temporal increases in the prevalence of autoimmune and allergic diseases reported in industrialized countries over the recent decades (Rodrigues et al. 2008). Changes in the environment associated with improved hygiene and cleanliness may therefore have important implications in the development of inflammatory diseases such as asthma, which are reaching epidemic levels in many Latin America countries, particularly among the urban poor.

In the present study, we investigated the effects of environmental exposures, many related to hygiene, on the expression of cytokines by unstimulated peripheral blood leukocytes in children 4–11 years of age living in poverty in the city of Salvador, in northeastern Brazil, where there is a large variation in the prevalence of various environmental exposures. Our novel data support the hypothesis that improvements in environmental hygiene may adversely affect immune homeostasis, as measured by the proportion of individuals spontaneously producing the regulatory cytokine interleukin (IL)-10. These data may also help to explain the increasing emergence of asthma, an inflammatory disease of dys-regulated immunity, as a major public health concern in urban areas.

The objective of the present study was to assess the impact of environmental exposures on immune homeostasis by measuring spontaneous cytokine production by unstimulated peripheral blood leukocytes in children living in urban Brazil.

## Materials and Methods

### Study population and data collection

The SCAALA (Social Change Asthma and Allergy in Latin America) Salvador Programme is conducted conducted in the city of Salvador, in northeastern Brazil, which has a population of 2.5 million. The prevalence of wheezing within the previous 12 months in this city in 2008 in schoolchildren 12–13 years of age is reported to be very high (27.1%) (Rodrigues et al. 2008). The design of this study has been reported elsewhere (Barreto et al. 2006; Rodrigues et al. 2008). In short, the study population included 1,376 children who were recruited in infancy for a prospective study measuring the impact of a citywide sanitation program on childhood morbidity (Barreto et al. 2007). We collected data from children born between 1994 and 2001 who lived in sentinel neighborhoods in the city. We applied standardized questionnaires to the children’s guardians between 1997 and 2003 (baseline) and included in our study data on demographic and social variables as well as an observation of the home environment (Strina et al. 2003). The children were surveyed again in 2005 to collect data on the same variables and to obtain blood samples. Of the 1,445 children included in the study, we assayed 1,006 for interferon-gamma (IFN-γ); 1,356 for IL-10; 1,289 for IL-13; and 1,243 for IL-5. For each analysis performed, we excluded any children without data for either the cytokine measurements or any study variable.

### Blood collection and whole-blood culture

We collected venous blood into heparinized tubes and cultured the collected cells at a dilution of 1:4 in RPMI (Gibco, Auckland, NZ) containing 10 mM glutamine (Sigma-Aldrich, St. Louis, MO, USA) and 100 μg/mL gentamicin (Sigma-Aldrich). The cells were cultured within 6 hr of collection and were maintained in a humidified environment of 5% CO_2_ at 37°C for 24 hr for detection of IL-10 and for 5 days for the detection of IL-13, IL-5, and IFN-γ in the presence or absence of pokeweed mitogen (Sigma-Aldrich). We determined the optimal time course of cytokine accumulation in whole-blood culture via a standardization process in our own laboratory.

### Cytokine production

We measured the spontaneous production of IL-5, IL-13, IFN-γ, and IL-10 in whole-blood culture supernatants using commercially available antibody pairs and recombinant cytokine standards (BD Biosciences Pharmingen, San Diego, CA, USA) by sandwich ELISA according to the manufacturer’s instructions. Cytokine concentrations were determined by interpolation of standard curves. The detection limit (lower/higher) of each cytokine was determined to be as follow: 15.63/500 pg/mL; 62.5/4,000 pg/mL; 18.5/300 pg/mL; and 31.25/500 pg/mL, respectively, for IL-5, IL-13, IFN-γ, and IL-10. We defined responders as children with cytokine concentrations above the lower limits of detection according to our assays (detectable results).

### Statistical analyses

We determined spontaneous cytokine production using geometric means and 95% confidence intervals (CIs), which were calculated by bootstrapping, that considered sex, age group (4–5, 6–7, 8–11 years of age), and breast-feeding status (< 2 months, 2–4 months, and ≥ 4 months) of the child. We also analyzed cytokine production according to socioeconomic characteristics, including maternal schooling, presence of tap water, status of garbage collection, and sewage system in the house. To correlate responsiveness of spontaneous cytokine production to the aforementioned variables, we used the Pearson chi-square test and trend test where appropriate. We calculated the odds ratios (ORs) and 95% CIs for measuring the strength of association between factors of interest and responsiveness of spontaneous cytokine production. We performed all statistical analyses using SPSS version 15.0 software (SPSS Inc., Chicago, IL, USA).

### Ethical considerations

We obtained ethical approval for this study from the Brazilian National Ethical Committee in 2004. Written informed consent was obtained from the legal guardian of each child.

## Results

### Description of study population

Table 1 contains the demographic characteristics of the study population as well as the environmental variables measured. We evaluated access to a household sewage system at two separate time points, early life (2000) and present time (2005). The study population age ranged from 4 to 11 years, and 40.4% of the population was between 6 and 7 years of age. Most of the children (77%) had been breast-fed (although not necessarily exclusively) for < 2 months (*n* = 786 breast-fed). Almost half (45.1%) of the mothers had completed a high school or college education. In total, 88.3% of the population had access to tap water, and 74.3% had daily or at least three times weekly garbage collection at the child’s home. With respect to a household sewage system, we obtained data from two different time points, including early in life when children were < 3 years of age as well as at the time of blood collection (late in life, 2005). Of the children who were surveyed, 13.8% did not have sewage system in either time period analyzed, 42.4% had a sewage system at one time point, and 43.8% had a sewage system in both time periods. It is important to clarify that in both periods the presence of a sewage system was defined as a household connected to the sewage system as stated by the child’s guardian.

### Spontaneous cytokine profile of studied children

**Figure 1:**
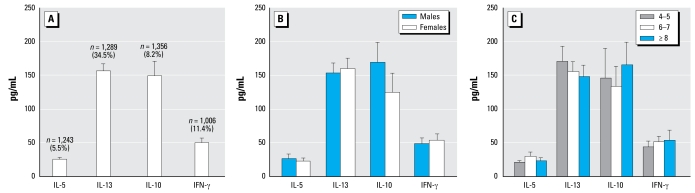
Cytokine production (pg/mL) overall (*A*), by sex (*B*), and by age (years) (*C*). Bars represent the geometric means, and vertical bars represent 95% CIs. The total sample size and percentage of responders are depicted at the top of each column.

About 11.4% (115 of 1,006) of the children were found to produce IFN-γ; 5.5% (68 of 1,243) IL-5; 8.2% (111 of 1,356) IL-10; and 34.5% (445 of 1,289) IL-13. The range of cytokine levels measured among responder children (i.e., excluding non-responder children from the analysis) is shown in Figure 1A. We also show the sex and age distributions of cytokine levels in Figures 1B and 1C, respectively. IL-10 and IL-13 were measured at the highest concentrations in culture (geometric mean: 149 pg/mL and 156.56 pg/mL, respectively). Both girls and boys produced similar levels of spontaneous cytokines, although there was some evidence for elevated levels of IL-10 in boys compared with girls. Spontaneous IL-3 production decreased with age, but no other age-dependent effects on cytokine levels were observed.

### Responsiveness of cytokine production according to biological characteristics and socioeconomic markers

Table 2 presents associations between cytokine production and important demographic and environmental parameters. We found no significant differences in T helper (Th) 1-type and 2-type cytokines production with respect to the children’s sex, although we did observe a tendency to produce more T regulatory (Treg)-type cytokine (IL-10) in males than in females (Table 2). Older children (6–7 and 8–11 years of age) seemed to produce more cytokines in culture than younger children. We observed that children who were breast-fed for > 4 months were more likely to be responders and produce spontaneous IFN-γ, IL-5, and IL-10 compared with children who were breast-fed for < 2 months; however, this difference was not statistically significant.

We found no significant associations between maternal education level or garbage collection in spontaneous cytokine production of population group. However, mothers with a higher educational level were more likely to have children with decreased spontaneous IL-10 production in culture, and children who lived in areas with inferior garbage collection produced more 2-type cytokines as well as Treg cytokine. A significant increase in IL-10 production was seen in those children whose households were not supplied with tap water (11.9% vs. 7.5%, χ^2^ *p* < 0.05). The chance of producing IL-10 is 67% greater for those children living in houses without a tap water supply compared with children whose households were supplied with tap water (OR =1.67; 95% CI, 1.05–2.64). With respect to a household’s sewage system, we did not find any differences between IL-13 (Th2-type cytokine) and IFN-γ (Th1-type) production in whole-blood cultures. On the other hand, IL-10 production was 8-fold higher (24.9%) in children without a sewage system in either time point compared with the group that had a sewage system in one time point (8.1%) or the group that always had a sewage system (3%) (*p* < 0.05, trend test). These data suggest that children who ever lived in households without a sewage system have a 10.76 times greater likelihood of producing IL-10 in culture than those who always lived in households with a sewage system (OR = 10.76; CI, 5.97–19.39). In addition, IL-5 production was significantly higher in children living in poor sanitation conditions (*p* < 0.05, trend test).

## Discussion

The aim of this study is to describe the cytokine profile of children between the ages of 4 and 11 years in a low-income population from a developing country according to sex, age, and environmental factors and exposures. These environmental parameters were characterized by maternal education level, breast-feeding, and the presence of adequate household garbage collection, sewage system, and tap water supply.

In this study, most of the subjects did not spontaneously produce cytokines, and the primary cytokines produced were IL-10 and IL-13. Resistance to most gastrointestinal nematodes is mediated by type-2 cytokine responses, in which IL-13 plays a dominant role. IL-13 is also an important cytokine mediator of allergic asthma and is responsible for regulating eosinophilic inflammation, mucus secretion, and airway hyperresponsiveness (Wynn 2003). On the other hand, the principal maintenance function of IL-10 appears to be limiting and ultimately terminating inflammatory responses. In addition to these activities, IL-10 also regulates the growth and/or differentiation of B cells, natural killer cells, cytotoxic and helper T cells, mast cells, granulocytes, dendritic cells, keratinocytes, and endothelial cells. IL-10 also plays a key role in the differentiation and function of a newly identified type of T cell, the Treg cell, which is an important cell type in the control of immune responses and tolerance *in vivo* (Moore et al. 2001). No significant difference was found in our study group in spontaneous cytokine production as a function of age, although we did see a trend in increasing IL-10 cytokine production in older children; this difference was not statistically significant. To our knowledge, no report has been published on spontaneous cytokine production in children as a function of sex. However, Eriksson et al. (2007) published a manuscript that compared the cytokine profiles of children and adults, reporting that children had fewer nondetectable measurements and more tumor necrosis factor-alpha (TNF-α) and IL-10 on lipopolysaccharide (LPS) stimulation, and adults seemed to produce more TNF-α, IL-5, and IFN-γ on phyto-hemaglutinin (PHA) stimulation. The same group also described that the production of cytokines TNF-α, IL-5, and IFN-γ was lower in girls than in boys in PHA-stimulated capillary blood cultures (Eriksson et al. 2007). According to our study, spontaneous IL-5, IL-13, and IFN-γ production was similar between boys and girls, but IL-10 levels were higher in boys; the differences were not statistically significant. This disparate result could be accounted for by different blood culture methods [whole blood cell (WBC) versus peripheral blood mononuclear cell culture] as well as by the stimulated versus unstimulated cells in culture.

We observed that children who were breast-fed for > 4 months spontaneously produced more detectable IFN-γ and IL-10 in whole-blood culture. Epidemiologic studies have indicated that breast-feeding is associated with infant health benefits and protects against many diseases, such as diarrhea (Victora et al. 1987), measles (Hanson 2007), pneumonia, and other respiratory tract infections (Cesar et al. 1999; Victora et al. 1987). Duramad et al. (2006) described a study in which children who were exclusively breast-fed (i.e., received no formula, cow’s milk, or solid foods) had significantly higher levels of 1-type cytokines at 1 month of age than infants who were not breast-fed. Because 1 is considered to protect against the development of asthma, these findings suggest a mechanistic explanation for the observed associations between breast-feeding and a decreased incidence of allergic pathology (Duramad et al. 2006; Guarner 2006; Matricardi and Bonini 2000). One potential mechanism to explain the effects of breast-feeding on immune disorders is that breast milk is implicated in aiding the establishment of native gut microbiota (acquired at birth and during the first year of life), which has been demonstrated to be an important modulator of the immune system (Guarner 2006). Penders et al. (2006) also reported that children who were breast-fed appeared to have the most beneficial gut microbiota (highest numbers of bifidobacteria and lowest numbers of *Clostridium difficile* and *Escherichia coli*). Bifidobacteria has also been implicated in protection against asthma and other allergic conditions (Tlaskalová-Hogenová et al. 2002).

Low maternal education levels are also associated with other socioeconomic variables, such as parameters related to household hygiene (Barreto et al. 2007). However, no significant differences were found in spontaneous cytokine production in association with maternal education, although we did see a trend in decreased IL-10 producers in whole-blood cell culture supernatant by children whose mothers had obtained higher education. In addition, a similar tendency was found in IL-10 production in children who lived in areas where garbage was collected three or fewer times per week.

With respect to the sewage system, our data indicate that children whose household had no sewage system at either time point when the data were collected (2000 and 2005) were more likely to produce IL-10 than those children in households with a sewage system in both 2000 and 2005 (Table 2; *p* < 0.05, trend test). Similarly, IL-10 production was increased in whole-blood cell cultures of children who did not have ready access to potable tap water (Table 2; χ^2^ *p* < 0.05). These data reflect the effects of environmental exposures in early life as well as on immune homeostasis in later childhood. Decreased hygiene (lack of access to clean drinking water and sanitation) in the first 3 years of life is associated with greater spontaneous IL-10 production up to 8 years later.

Exposure to pathogens and their products, and helminths in particular, has been associated with poor sanitation such as an absence of an adequate sewage system or access to tap water (Hagel et al. 2008). In agreement with this observation, our data also revealed that children who live in areas both without a sewage system (2000–2005) as well as access to tap water display a greater frequency of *Ascaris lumbricoides* and *Trichuris trichiura* infections (data not shown; χ^2^ *p* < 0.05). This suggests that exposure of the body to helminth infections may play an important role in modulating the immune response. Helminth infections and their effects on the immune system have been characterized to include a strong upregulation of Treg cells, increased production of IL-10 (Maizels and Yazdanbakhsh 2003; Wilson et al. 2005), and, in this study, an increased percentage IL-10 responders in children without ready access to a sewage system or tap water can be partially explained by this higher prevalence of helminth infections (data not shown; χ^2^ *p* < 0.05). Although helminth infections induce strong Th2 responses, adult worms are often able to survive in the human host, sometimes even for decades. Survival of the adult worms is thought to be facilitated by the induction of an immune regulatory network. These regulatory mechanisms include modulation of the innate immune system including macrophages and dendritic cells, and induction of regulatory T cells promoting an anti-inflammatory environment (Yazdanbakhsh et al. 2002). This response is characterized by increased concentrations of IL-10 and transforming growth factor β (TGF-β). This regulatory network prevents the elimination of the worms but at the same time protects the host against pathology that would otherwise result from excessive inflammation (Maizels and Yazdanbakhsh 2003; Wilson et al. 2005). This hyporesponsiveness is not only directed toward parasite antigens, but also appears to extend to third-party antigens. For example, chronic infection with different types of helminths has been shown to reduce the responsiveness of the immune system to the tetanus vaccine (Cooper et al. 1998; Nookala et al. 2004; Sabin et al. 1996). There is also evidence in the literature that development of autoimmune and allergic pathologies may be modified by helminth infections (Akbari et al. 2002; Barrat et al. 2002; Burkhart et al. 1999; Green et al. 2003; Smits and Yazdanbakhsh 2007; Zhang et al. 2004). Turner et al. (2008) demonstrated that in helminth hyperendemic regions, estimators of combined intestinal nematode infections were positively associated with spontaneous IL-10 and TGF-β1 production and negatively associated with total immune reactivity, suggesting that gut nematodes are important mediators of immunoregulation. Treg cell IL-10, TGF-β producers, immunoglobulin G4 isotype-blocking antibodies, and suppressed mast cells, basophils, and eosinophils appear to be the major components of a healthy immune response against aeroallergens (Akdis et al. 2008).

In addition to helminths, other microorganism components have also been described to upregulate IL-10, including fungal components (Carvalho et al. 2002; Jeurink et al. 2008), bacteria (Oderda et al. 2007), and protozoa (Prigione et al. 1995). In particular, microorganisms assembled in the ecologic niche of a host individual, termed microbiota, also seem to play an important role in modulation of the immune system (Guarner 2006). Microbiota are continuously ingested from the environment (e.g., food, beverage), and the genetic background of the individual appears to play a role in determining the commensal microbiota profile (Khachatryan et al. 2008). Abnormalities in the development of the immune system may be attributable partly to defects in the interaction of the flora with mucosal immune compartments (Tlaskalova-Hogenova et al. 2004). According to the hygiene hypothesis, an increased incidence of allergic pathology in westernized societies may be explained in part by a reduced microbial load early in infancy (Rook and Brunet 2002). In this context, an appropriate composition of commensal flora is necessary. Exposure to food-borne and orofecal, nonpathogenic microbes most likely helps to exert a homeostatic impact (Guarner 2006) as long as living, multiplying bacteria and also their components—including LPS, superantigens, peptidoglycans, bacterial CpG–DNA motifs, and heat shock proteins, which are expressed, secreted, and potentially released from the bacteria after microbial death, are present. These factors are responsible for various immunomodulatory effects induced by innate immunity (toll-like receptors) as well as regulatory network activation (Baba et al. 2008; Tlaskalova-Hogenova et al. 2004), which may also reflect protection against allergic and autoimmune diseases (Guarner 2006).

The low-income population represented in our study presents a high prevalence of asthma and other allergic diseases (Rodrigues et al. 2008). We have demonstrated that the absence of a sewage system or tap water is associated with an upregulation of IL-5 and IL-10 production in whole-blood culture. As a result of these findings, we are now interested in investigating which exposures (e.g., viruses such as hepatitis A, Epstein–Barr, and herpes zoster; bacteria such as *Helicobacter pylori* and commensal microbiota; and parasites such as *Ascaris lumbricoides* and *Trichuris trichiura*) are involved in this spontaneous and antigen-stimulated blood cell immune profile. Importantly, we would like to understand the consequences of this immune modulation with respect to the development of asthma/atopy and other allergic reactions.

## Figures and Tables

**Table 1:** Biological and socioeconomic characteristics of children in Social Change Asthma and Allergy in Latin America Group (SCAALA).

Variable	No. (%)
Sex	

Male	743 (54.0)
Female	633 (46.0)

Age (years)	

4–5	356 (25.9)
6–7	556 (40.4)
8–11	464 (33.7)

Breast-feeding period (months) (*n* = 786)	

< 2	605 (77.0)
2–4	93 (11.8)
≥ 4	88 (11.2)

Maternal education	

Elementary school	301 (22.2)
Middle school	443 (32.7)
High school or college	612 (45.1)

Tap water	

No	228 (16.7)
Yes	1,136 (83.3)

Garbage collection	

Daily or at least 3 days/week	994 (74.7)
< 3 day/week	336 (25.3)

Sewage system (in 2000 and 2005)	

Never	183 (13.8)
At least in one time point	561 (42.4)
Always	580 (43.8)

**Table 2:** Responsiveness to spontaneous cytokine production according to biological and socioeconomic characteristics.

	IFN- γ				IL-5				IL-13				IL-10			
Variables	No.	Percent response	OR	(95% CI)	No.	Percent response	OR	(95% CI)	No.	Percent response	OR	(95% CI)	No.	Percent response	OR	(95% CI)
Overall	1,006	11.4	—	—	1,243	5.5	—	—	1,289	34.5	—	—	1,356	8.2	—	—

Sex																

Female	472	10.2	1.00		575	5.9	1.00		597	34.8	1.00		627	7.3	1.00	
Male	534	12.5	1.27	(0.86–1.88)	668	5.1	0.85	(0.52–1.39)	692	34.2	0.97	(0.77–1.23)	729	8.9	1.24	(0.83–1.83)

Age (years)																

4–5	256	9.4	1.00		322	4.0	1.00		334	32.9	1.00		351	7.4	1.00	
6–7	413	13.8	1.55	(0.93–2.56)	499	6.0	1.52	(0.78–2.96)	525	34.5	1.07	(0.80–1.43)	543	7.4	0.99	(0.60–1.66)
8–11	337	10.1	1.08	(0.63–1.88)	422	5.9	1.50	(0.75–2.97)	430	35.8	1.14	(0.84–1.54)	462	9.7	1.35	(0.81–2.23)

Breast-feeding period (months)^a^																

< 2	457	9.6	1.00		542	3.9	1.00		575	35.7	1.00		605	8.3	1.00	
2–4	72	13.9	1.51	(0.72–3.16)	88	5.7	1.49	(0.55–4.07)	90	33.3	0.90	(0.56–1.44)	93	11.8	1.49	(0.74–2.98)
≥ 4	55	12.7	1.37	(0.58–3.20)	82	6.1	1.61	(0.59–4.40)	84	34.5	0.95	(0.59–1.54)	88	11.4	1.42	(0.69–2.92)

Maternal education																

Elementary school	212	11.3	1.00		274	4.7	1.00		281	33.1	1.00		299	10.4	1.00	
Middle school	333	12.6	1.13	(0.66–1.93)	403	5.0	1.05	(0.51–2.14)	417	35.7	1.12	(0.82–1.55)	436	8.0	0.75	(0.45–1.25)
High school or college	447	11.0	0.96	(0.57–1.62)	548	6.0	1.29	(0.67–2.49)	571	34.3	1.06	(0.78–1.43)	601	7.3	0.68	(0.42–1.11)

Tap water																

Yes	829	10.9	1.00		1,033	5.6	1.00		1,066	34.8	1.00		1,118	7.5	1.00	
No	165	13.9	1.33	(0.81–2.18)	198	5.1	0.89	(0.45–1.78)	213	33.8	0.96	(0.70–1.30)	226	11.9*	1.67	(1.05–2.64)

Garbage collection																

Daily or at least 3 days/week	239	13.0	1.00		301	4.3	1.00		310	33.2	1.00		335	6.9	1.00	
<3 day s/week	730	10.5	0.79	(0.51–1.23)	899	5.7	1.33	(0.71–2.49)	941	34.8	1.08	(0.81–1.41)	975	8.8	1.32	(0.32–1.39)

Sewage system																

Always	411	11.7	1.00		526	4.0	1.00		543	32.8	1.00		570	3.0	1.00	
At least in one time point	415	9.9	0.83	(0.53–1.29)	513	7.0	1.86	(0.88–3.06)	523	35.8	1.14	(0.89–1.47)	553	8.1	2.88	(1.63–5.10)
Never	144	13.9	1.22	(0.70–2.14)	153	7.2**	1.82	(1.04–3.15)	174	32.2	0.97	(0.68–1.40)	181	24.9**	10.76	(5.97–19.39)

a *n* = 786.

* *p* < 0.05 (χ^2^ test).

** *p* < 0.05 (trend test).
